# Malleostapedotomy in Patients With Stapes Fixation: A Systematic Review

**DOI:** 10.1002/lary.70232

**Published:** 2025-11-13

**Authors:** Matteo Alicandri‐Ciufelli, Edoardo D'Alessandro, Daniela Lucidi, Riccardo Nocini, Hui Davide Qiu

**Affiliations:** ^1^ FACS, FEBORL‐HNS, ENT Department at Policlinico di Modena University of Modena and Reggio Emilia Modena Italy; ^2^ ENT Department at Policlinico di Modena University of Modena and Reggio Emilia Modena Italy; ^3^ UOC Otorinolaringoiatria—Università Degli Studi di Bologna—AUSL Romagna—Sede di Ravenna Ravenna Italy; ^4^ Unit of Otolaryngology, Head and Neck Department University of Verona Verona Italy

**Keywords:** malleostapedopexy, malleostapedotomy, otosclerosis, vestibulomalleopexy

## Abstract

**Objective:**

To review the literature regarding malleostapedotomy as primary or revision surgery in patients with stapes fixation, to point out the state of the art regarding indications, techniques and outcomes of this procedure.

**Data Sources:**

PubMed, CINAHL and Cochrane databases were screened following the Preferred Reporting Items for Systematic Reviews and Meta‐Analyses guidelines.

**Review Methods:**

Clinical studies describing malleostapedotomy in patients with stapes fixation were included in this Systematic Review. Data about indications, intraoperative findings, features of the prostheses and audiological results were recorded.

**Results:**

A total of 25 articles and 632 ears that underwent malleostapedotomy were included in the analysis. This technique has been employed for both primary and revision surgeries, evolving over time alongside advancements in the prostheses used. Revision otosclerosis surgery was identified as the most common surgery necessitating malleostapedotomy. Among the cases, 44.8% achieved ABG closure within 10 dB, while 84.9% achieved ABG closure within 20 dB.

**Conclusions:**

Malleostapedotomy is a safe and valid surgical technique that can be performed when incus anchoring stapedoplasty is not feasible.

## Graphical abstract

This systematic review analyses the current evidence on malleostapedotomy as a primary or revision procedure in patients with stapes fixation. Twenty‐five studies comprising 632 operated ears were included. Malleostapedotomy proved to be a safe and effective alternative to incus‐anchoring stapedoplasty, with favorable hearing outcomes and a low complication rate.

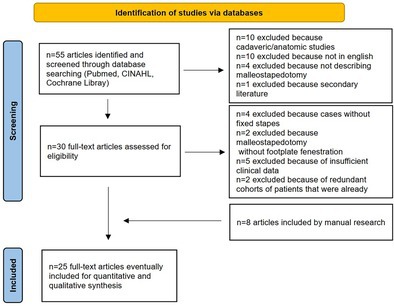

## 1 Introduction

Malleostapedotomy (MS) originated as an incus‐bypass technique for stapes footplate fixation when the incus was absent, eroded, or unsuitable. Early concepts emerged in the 1950s and were developed in the 1970s–1980s by Sheehy [1] and by Schuknecht and Bartley [2], who used stainless‐steel or Teflon‐wire prostheses. Their transcanal method required wide elevation of the tympanic membrane and crimping a wire loop around the malleus handle. Although these series showed good closure of the air–bone gap, the procedure was technically demanding and inner‐ear complications were not rare, so it was reserved for a small minority of stapes operations.

In the late 1980s Ugo Fisch and co‐workers reintroduced the operation under the term “malleostapedotomy” [3]. They proposed three critical improvements: an endaural incision with partial anterosuperior canalplasty for better exposure of the upper malleus and anterior malleal ligament; limited elevation of the tympanic membrane with the prosthesis loop crimped near the lateral malleal process to reduce the large physiological movements of the umbo; and a dedicated 8.5 × 0.4 mm titanium piston that could be bent to maintain a perpendicular orientation on the footplate and resist long‐term malleus motion. These changes reduced the risk of prosthesis displacement and inner‐ear trauma and made the surgery more reproducible.

During the 1990s and 2000s further refinements consolidated the technique: the use of self‐crimping or nitinol pistons (Häusler [4]; Magliulo [5]), endoscopic visualization (Iannella [6]; Son [7]), and adaptation for complex revision cases (Rambousek [8]; Gargula [9]). As a result, MS evolved from a rare salvage option to an accepted alternative to incudostapedotomy (IS) for advanced otosclerosis, congenital ossicular malformations, and revision surgery after failed stapes procedures.

This systematic review aims to address the following question: in patients with stapes fixation, is MS a safe and effective surgical technique in terms of audiological outcomes and complications, both in primary and revision procedures?

## 2 Methods

This systematic review has been reported according to PRISMA 2020 guidelines.

A structured search of the literature was performed in March 2025 using 3 major databases, CINAHL, PUBMED, Cochrane Library, with the following search terms: “malleostapedotomy” OR “malleovestibulopexy” OR “malleostapedopexy” OR “malleostapedoplasty” OR “malleostapedoplastic” OR “malleostapedectomy”. After running the above search terms, abstracts and titles were obtained. All the titles and abstracts extracted from this research have been read and evaluated independently by two of the authors (HDQ and ED) to identify the list of eligible citations. Disagreements on some citations to be included have been discussed with an expert (MAC).

The eligibility criteria for study inclusion were based on the following PICO framework:

- Population (P): patients with stapes fixation undergoing middle ear surgery (both primary and revision procedures).
- Intervention (I): MS—a surgical technique involving anchoring the prosthesis to the malleus.
- Comparison (C): IS or other traditional stapedoplasty techniques, or no direct comparison (in studies lacking a control group).
- Outcomes (O): audiological outcomes (e.g., air‐bone gap closure), complication rates, surgical indications, feasibility and safety of the technique.
- Study design (S): systematic review.

Inclusion criteria for citations were:

- english language;
- article describing indications, technique and result of MS.

Exclusion criteria were:

- cadaveric or merely anatomic studies;
- second literature articles.

The full texts of the articles identified were obtained for a second screening, in order to select studies for inclusion.

Inclusion criteria for full‐text articles identified were:

- presence of fixation of stapes;
- presence of hearing outcomes;
- the use of a prosthesis anchored to the malleus and inserted into the vestibule through a fenestration in the stapes footplate;
- follow‐up at least of 3 months.

Exclusion criteria for full‐text articles identified were:

- lack of sufficient clinical data;
- redundant cohorts of patients that were already reported by the same authors.

A further manual check was performed on the references included within articles.

Full‐text articles were independently reviewed in their entirety by two authors (E.D. and H.D.Q.). Any discrepancies regarding study eligibility were resolved through consensus, and, when necessary, by consultation with an expert (MAC). Decisions on study inclusion or exclusion were consistently guided solely by the prespecified eligibility criteria.

The protocol for this systematic review was not registered in a publicly accessible database. Although registration is recommended it is not mandatory according to PRISMA 2020; all methodological steps were predefined and rigorously followed to ensure transparency and minimize the risk of selective reporting.

The same two authors (E.D. and H.D.Q.) proceeded independently to extract data and report it on an Excel file.

In the case of studies including different groups other than patients undergoing MS procedures, only the MS group was considered and included in this review. Studies in which it was not possible to separately extract the data for the MS group from the other groups in the study were excluded.

Data about demographics, kind of prosthesis used, primary/revision surgeries, underlying diagnoses, intraoperative findings, audiometric records, type and dimensions of prosthesis and post operative complications were recorded; subsequently qualitative and quantitative synthesis was performed.

Due to the substantial heterogeneity in study design, surgical techniques, prostheses, and outcome reporting, a formal meta‐analysis was not feasible. For the same reasons, subgroup analyses could not be reliably performed. Therefore, results are presented descriptively, with percentages calculated for illustrative purposes only, without statistical pooling.

A dedicated database was created to collect patient and outcome data from the included studies. Results were extracted as reported by the authors, either in absolute numbers or as percentages. Most studies expressed hearing outcomes in terms of air–bone gap (ABG) closure within 10 dB or within 20 dB, while only a few reported mean ABG values. When information was incomplete, missing values were calculated manually only if the data provided in the full text or tables allowed for unambiguous reconstruction, without altering the original results. If data could not be clearly derived, they were not imputed. All percentages presented in the review were therefore calculated on the number of patients for whom the relevant variable was available. Percentages of ABG closure (e.g., < 10 dB or < 20 dB) were calculated as weighted averages, taking into account the number of patients reported in each study, rather than as simple arithmetic means across studies.

Risk of bias was assessed for all included studies using a modified version of the Newcastle–Ottawa Scale (NOS), adapted to the characteristics of retrospective observational case series, prospective cohorts, and case reports. The modified tool (File S1) evaluated three main domains: (1) selection (0–4 points: representativeness of the cohort, clarity of inclusion criteria, use of standardized diagnostic methodology, adequacy of sample size); (2) comparability (0–2 points: control of relevant clinical variables, use of comparable groups or clearly defined subgroups); and (3) Outcome/Exposure (0–3 points: definition and standardization of outcome assessment, adequacy and reporting of follow‐up, description and handling of losses to follow‐up).

Each criterion was scored 0–1, for a maximum total score of 9 points. Studies were classified as low risk of bias (7–9 points), moderate risk (4–6 points), or high risk (0–3 points).

## 3 Results

**FIGURE 1:**
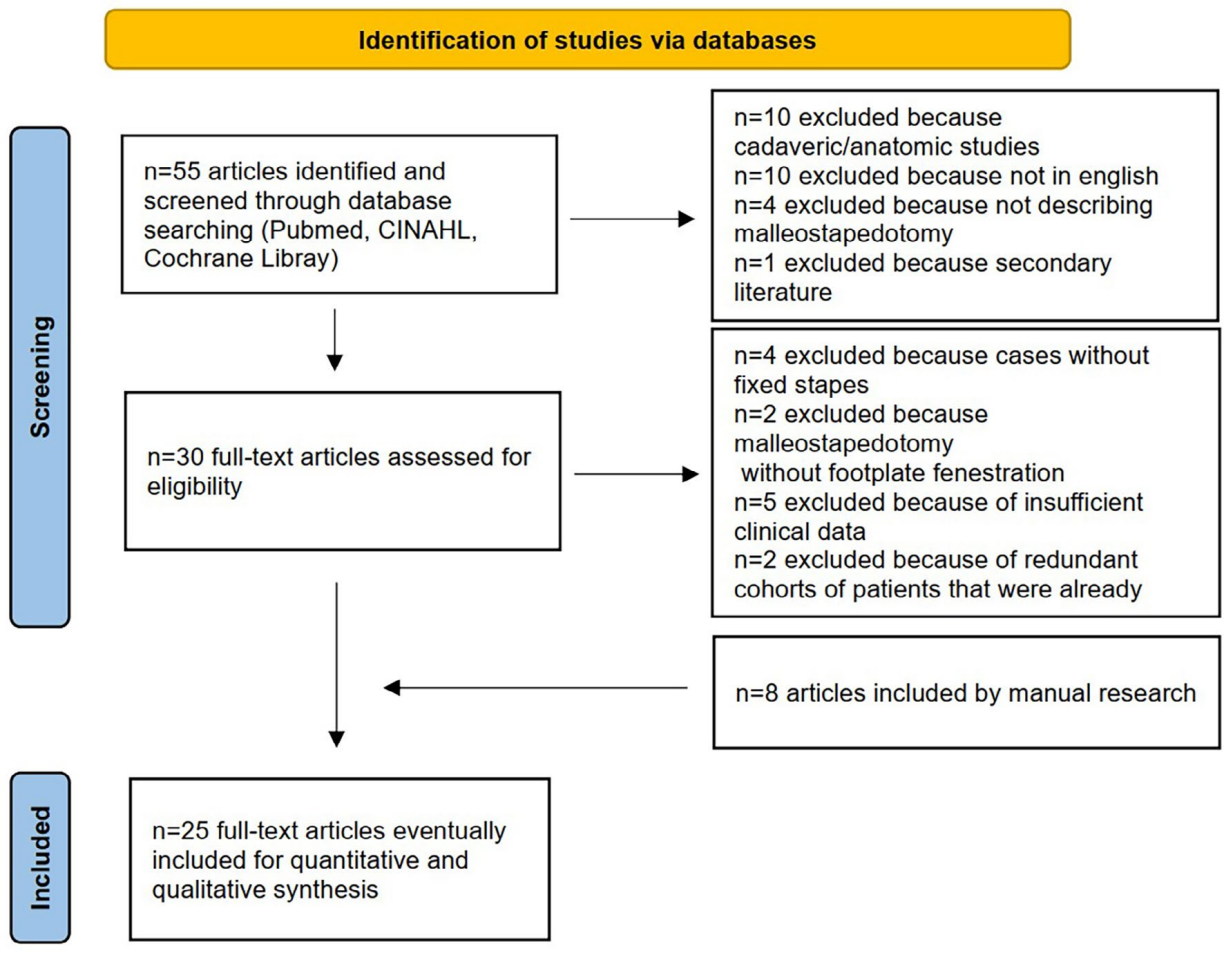
PRISMA 2020 flowchart of study selection. [Color figure can be viewed in the online issue, which is available at www.laryngoscope.com]

Running the above search string in 3 major databases (CINAHL, PubMed and Cochrane Library), 55 articles were identified till March 2025. After an initial check, full‐text retrieval and manual cross‐checking of references included within the articles, 25 studies published between 1986 and 2024 clearly met the inclusion criteria and were chosen for the analysis (Figure 1). Table 1 summarizes the main characteristics of the included studies. Table 2 provides a summary of the patients' characteristics and intraoperative findings reported across the included studies. Please note that the reported percentages in this section are based solely on the number of patients for whom the corresponding information was available, without statistical pooling across studies.

**TABLE 1:** Characteristics of the included studies.

Nos	Authors	Year	Study type	Number of ears undergone to malleostapedotomy	Type of surgery	Surgery technique	Etiologies (*n* (%))	Prosthesis type	Prosthesis lenght	Intraoperative findings (*n* (%))	Preoperative hearing PTA ABG (mean; dB) (min–max)	Postoperative PTA ABG (dB) (%)	Postoperative complications (*n* (%))
1	Rambousek [8]	2012	Prospective	60	28 primary 32 revision	Microscopic	Primary: Oto 20 (71) Tymp 3 (11) Chol 2 (7) CM 2 (7) Other 1 (4) Revision: Oto 21 (66) Tymp 4 (13) Chol 2 (6) Other 5 (15)	0.4 mm Fisch titanium stapes piston	Mean 7 mm (6–8.25 mm)	Primary: Lat 21 (75) Inc 5 (18) IMD 1 (4) Revision: Lat 6 (19) Inc 12 (38) IMD 3 (9) Dis 19	Primary 26.3 Revision 27.5	Primary mean 9.4 (< 10 dB 61%, < 20 dB 100%) Revision mean 11.3 (< 10 dB 38%, < 20 dB 81%)	SNHL 2 (3)
2	Thomeer [10]	2011	Retrospective	12	N/A	Microscopic	CM 12 (100)	Fisch Teflon‐platinum piston	N/A	Lat 12 (100)	40 (14–65)	< 10 dB 33%, < 20 dB 83%, > 20 dB 17%	N/A
3	Dalchow [11]	2007	Case series	6	4 primary 2 revision	Microscopic	Oto 6 (100)	0.4 mm titanium piston	Mean 6.8 mm (6.3–7.5 mm)	Primary Lat 4 Revision Inc 2	N/A	< 10 dB 4 (66.6%), 11–20 dB 1 (16.6%), 21–30 dB 1 (16.6%)	None
4	Magliulo [12]	2007	Prospective	10	N/A	Microscopic	Tymp 10 (100)	50% Fluoroplastic piston 50% Nitinol piston	N/A	Lat 4 Inc 3	N/A	< 10 dB 2 (20%), 11–20 dB 5 (50%), 21–30 dB 2 (20%), > 31 dB 1 (10%)	SNHL 1 (10)
5	Fisch [3]	2001	Retrospective	56	Revision	Microscopic	Oto 56 (100)	84% 0.4 mm platinum‐ribbon Teflon piston 16% 0.4 mm titanium piston	Mean 6.5 mm (5.5–7.0 mm)	N/A	N/A	< 10 dB 10 (18%), 11–20 dB 33 (59%), 21–30 dB 8 (14%), > 31 dB 5 (9%)	SNHL 2 (3)
6	Son [7]	2024	Retrospective	7	N/A	Endoscopic	Oto 3 (43) CM 4 (57)	Platinum/fluoroplastic piston or McGee‐modified loop piston	N/A	N/A	58.8	Mean 10.7 (< 10 dB 2 (28.6%), 11–20 dB 5 (71.4%))	Taste 1 (14)
7	Alabdulqader [13]	2021	Case series	2	Revision	Microscopic	Oto 1 (50)	0.4 mm Causse Looperative piston	6 mm	IMD 2 (100) (iatrogenic)	40	Mean 7.5 (< 10 dB 1 (50%), 11–20 dB 1 (50%))	None
8	Gargula [9]	2020	Retrospective	12	2 Primary 10 Revision	Microscopic	Oto 12 (100)	0.6 mm Nitinol SMart malleus to footplate piston	6 mm–6.5 mm	Lat 2 (17) Inc 10 (83)	31.4 (15–55)	Mean 7.45 (< 10 dB 9 (75%), 11–20 dB 2 (16.7%), 21–30 dB 1 (8.3%))	None
9	Burggraaf [14]	2018	Retrospective	16	2 Primary 14 Revision	Microscopic	Oto 11 (69) Tymp 4 (25) Other 1 (6)	0.4 mm or 0.6 mm CliP Piston MVP Häusler Design	Mean 6.25 mm (5.75–6.5 mm)	N/A	41.4 (24–60)	Mean 17.3 (< 10 dB 5 (31, 2%), 11–20 dB 7 (43.7%), 21–30 dB 1 (6.25%), > 30 dB 2 (12.5%))	Disp 2 (12.5)
10	Iannella [6]	2018	Case series	6	Revision	Endoscopic	Oto 6 (100)	0.4 mm or 0.5 mm superelastic nitinol piston (Audio Technologies)	4.25–7.0 mm	Lat 6 (100)	35	Mean 14 (< 10 dB 2 (33, 3%), 11–20 dB 3 (50%), > 30 dB 1 (16.6%))	Taste 2 (33.3)
11	Park [15]	2014	Retrospective	35 (20 handle‐MS; 15 neck‐MS)	27 Primary 8 Revision	Microscopic	Oto 15 (43) Tymp 1 (3) CM 19 (54)	0.4 mm platinum‐wire‐piston (PWP)	6 mm	Lat 15 (43) Inc 18 (51) IMD 5 (14)	N/A	sss (31.4%), 11–20 dB 12 (34.3%), 21–30 dB 9 (25.7%), > 30 dB 3 (8.6) Handle‐MS < 10 dB 6 (30%), 11–20 dB 5 (25%), 21–30 dB 8 (40%), > 30 dB 1 (5%) Neck‐MS < 10 dB 5 (33.3%), 11–20 dB 7 (46.7%), 21–30 dB 1 (6.7%), > 30 dB 2 (13.3%)	Disp 1 (3)
12	Magliulo [5]	2013	Prospective	14	N/A	Microscopic	N/A	0.4 mm self crimping‐polytetrafluoroethylene (SC‐PTFE) prosthesis	N/A	Lat 8 (57) Inc 4 (29) IMD 2 (14)	N/A	< 10 dB 5 (35.7%), 11–20 dB 6 (42.9%), 21–30 dB 2 (14.3%), > 30 dB 1 (7.1%)	SNHL 1 (7)
13	Schuknecht [2]	1986	Retrospective	203	N/A	Microscopic	Oto 138 (68)	Fat‐wire prosthesis Teflon‐wire prosthesis Wire only prosthesis Gelfoam‐wire piston Steel piston	N/A	Lat 45 Inc 65 IMD 14	36	Mean 14 (< 15 dB 52%, 16–20 dB 15.5%, > 20 dB 32.5%)	SNHL 16 (8.4) Disp 14 (6.9)
14	Tange [16]	1996	Retrospective	41	1 Primary 40 Revision	Microscopic	N/A	Teflon malleus attachment piston	N/A	Lat 11 (27) Inc 17 (41)	N/A	Mean 20 (< 10 dB 29 (70.7%), < 20 dB 36 (87.8%), > 30 dB 3 (7.3%))	Disp 2 (4.8)
15	Häusler [17]	2004	Retrospective	44	13 Primary 31 Revision	Microscopic	N/A	0.6 or 0.8 mm Teflon piston–metal wire prosthesis or modified malleus handle (malleus grip) prosthesis	5.0–6.5 mm	Lat 17 Inc 7 IMD 6	N/A	< 10 dB 40%, < 20 dB 85%	SNHL 3 (7) Disp 1 (2)
16	Sarac [18]	2006	Retrospective	36	Revision	Microscopic	Oto 36 (100)	0.4, 0.6, 0.8 mm Schuknecht malleus grip prosthesis	mean 5.5 mm (5.5–6.0 mm)	Lat 3 (8) Inc 30 (83) IMD 3 (8)	43.3	Mean 14.3 (< 10 dB 16 (44%), < 20 dB 26 (72%))	SNHL 2 (6) Disp 3 (9) Dizz 1 (3)
17	Kohan [19]	2003	Retrospective	5	Revision	Microscopic	N/A	Wire Teflon‐piston	N/A	Inc 80% Dis 73%	29 (17–54)	Mean 7 (< 10 dB 3 (60%), 11–20 dB 2 (40%))	None
18	Xu [20]	2020	Retrospective	10	6 Primary 4 Revision	Microscopic	Oto 7 (70) Tymp 3 (30)	0.6 mm Self‐Crimping MS piston	6.5–7.5 mm	Lat 4 (40) Inc 4 (40) IMD 2 (20)	37.3 (23.75–63.75)	Mean 12.5 (< 10 dB 4 (40%), 11–20 dB 5 (50%), > 30 dB 1 (10%))	Disp 1 (10)
19	Pitiot [21]	2016	Retrospective	10	Revision	Microscopic	Oto 10 (100)	Teflon piston loop	N/A	N/A	N/A	< 10 dB 5 (50%), 11–20 dB 4 (40%), > 20 dB 1 (10%)	None
20	Van Rompaey [22]	2011	Retrospective	10	Revision	Microscopic	Oto 10 (100)	0.4 or 0.6 mm Teflon piston	N/A	N/A	N/A	< 10 dB 50%, < 20 dB 90%	None
21	Häusler [4]	2007	Case series	10	2 Primary 8 Revision	Microscopic	Oto 8 (80) CM 2 (20)	0.4 mm or 0.6 mm CliP Piston MVP Häusler Design	5–6.25 mm	Lat 2 (20) Inc 8 (80)	N/A	< 20 dB 100%	Disp 1 (10)
22	Kisilevsky [23]	2009	Retrospective	24	7 Primary 17 Revision	Microscopic	Oto 17 (71) CM 7 (29)	Modified Cawthorne (0.3 mm diameter) or modified Causse (0.4 mm diameter) prosthesis	5–6 mm	N/A	33	Mean 20 (< 10 dB 33%, < 20 dB 61%)	None
23	van der Rijt [24]	2003	Case series	1	Revision	Microscopic	Other 1 (100)	0.4 mm Cremers modified Fish Teflon‐platinum piston	5.5 mm	IMD 1	N/A	17 dB	N/A
24	De Leenheer [25]	2003	Case report	1	Primary	Microscopic	Other 1 (100)	Cremers modified Fish Teflon‐platinum piston	5 mm	Inc 1 (100)	N/A	0 dB	N/A
25	Ensink [26]	1997	Case report	1	Primary	Microscopic	Other 1 (100)	0.4 mm teflon‐platinum piston	4.75 mm	Lat 1	N/A	15 dB	N/A

Abbreviations: ABG: air‐bone gap; Chol, cholesteatoma; CM congenital malformation; Dis, displaced prosthesis; Disp, prosthesis displacement/extrusion; Dizz, persistent dizziness; IMD, incudomalleolar dislocation; Inc, incus eroded/necrosis/absent; Lat, later ossicular chain fixation; Other, other (acquired ossicular fixation, tumor, osteogenesis imperfecta); Oto, otosclerosis; PTA: Pure tone audiometry; SNHL, sensorineural hearing loss; Taste, abnormale taste sensation; Tymp, tympanosclerosis.

**TABLE 2:** Patient characteristics across the included studies.

Total no of reported malleostapedotomy	632
Type of surgery	% of 386 pts. with reported type of surgery
Primary	24.4%
Revision	75.6%
Underlying etiologies	% of 528 pts. with reported etiologies
Cholesteatoma	0.8%
Other (acquired ossicular fixation, tumor, osteogenesis imperfecta, syndromic conditions)	1.7%
Tympanosclerosis	4.7%
Congenital malformation of the middle ear	8.7%
Otosclerosis	71.4%
Intraoperative findings	% of 509 pts. with reported intraoperative findings
Displaced prosthesis	3.7%
Incudomalleolar dislocation	5.5%
Lateral ossicular chain fixation	28.3%
Incus eroded/malformed/necrosis/absent	35.1%
Complications	% of 617 pts. with reported complications
Anacusis	0.0%
Persistent vertigo	0.2%
Hypogeusia	0.5%
Prosthesis extrusion/misplacement	4.1%
Sensorineural hearing loss	4.4%

*Note*: Data are presented in four sections: Type of surgery (primary vs. revision), underlying etiology, intraoperative findings, and postoperative complications. Percentages were calculated using as denominator only the number of patients for whom the respective variable was explicitly reported, and therefore may differ across sections. In the etiology section, totals do not sum to 100% because some studies grouped multiple etiologies together in the same category, preventing a precise stratification of individual diagnoses.

Most of the selected articles are retrospective studies. The largest series, reported by H.F. Schuknecht et al. [2], included 203 cases and described middle ear surgery MS outcomes using various malleus‐anchoring prostheses. A total of 632 patients who underwent MS were included. Among the cases where the type of surgery was specified, approximately 25% were primary procedures and 75% were revisions. Revision cases mostly involved pathologies such as otosclerosis, while primary surgery has mainly been performed in patients with congenital malformations of the middle ear. The most used surgical technique was microscopic, with only two authors, Son et al. [7] and Iannella et al. [6], reporting a series of endoscopic MS procedures.

Regarding the underlying diagnosis for the procedures, otosclerosis was the most frequent condition, with an average rate of 66.3%, followed by congenital middle ear malformations (stapes fixation associated with lateral chain fixation) with an average rate of 17.8%. Other reported conditions included tympanosclerosis, cholesteatoma, neoplastic diseases, trauma, osteogenesis imperfecta and syndromic conditions involving the middle ear.

When selecting the stapes prosthesis to be attached to the malleus, the literature reported the use of various prostheses differing in material, shape, and size. The most common piston diameter was 0.4 mm, while prostheses were described in different materials, including steel, platinum, Teflon, or nitinol. The length of the prostheses used in the examined sample, usually determined based on intraoperative findings and measurements, ranged from 4.25 to 8.25 mm. Furthermore, the prosthesis was often reshaped to achieve an adequate angle between the oval window and the handle of the malleus. Unlike other authors who described the classical MS technique with a prosthesis attached to the handle of the malleus, Park proposed a technique with the prosthesis anchored to the neck of the malleus [15].

Intraoperative findings are reported in Table 1 and were categorized into four main groups: fixation of the lateral ossicular chain, absence/erosion/necrosis of the incus, dislocation of the incudomalleolar joint and extrusion/misplacement of the prosthesis.

The average preoperative air‐bone gap (ABG) pure‐tone audiometry (PTA) value across the pooled patient cohort was 39.75 dB.

The success of the procedure is determined based on PTA ABG closure, classified as excellent if < 10 dB and good if < 20 dB.

An excellent result was achieved in 40.4% of cases, while a good result was achieved in 82.4% of cases.

Complications were explicitly reported for 617 out of the 632 surgical cases. Within this cohort, 27 instances of postoperative sensorineural hearing loss were documented (4.4%), although no cases of anacusis were observed. Late prosthesis extrusion or misplacement occurred in 25 cases (4.1%). Persistent vertigo lasting more than 3 weeks was reported in one patient (0.2%), while three cases of hypogeusia related to chorda tympani nerve injury were described (0.5%).

A qualitative assessment of bias was performed for all included studies, the majority of which were retrospective observational case series. Overall, most studies were judged at moderate to high risk of bias. Selection bias may have been introduced, as patients were often included based on intraoperative findings without standardized inclusion criteria; only a minority reported consecutive inclusion or clearly defined diagnostic protocols. Performance bias was evident due to heterogeneity in surgical techniques, prosthesis types, and surgeon experience. Detection bias arose from inconsistent reporting of audiological outcomes, with variability in frequency ranges, follow‐up durations (6 weeks to 12 months), and definitions of air–bone gap (ABG) closure. Attrition bias was present, as dropout rates and follow‐up losses were rarely detailed. Reporting bias was also a concern, given inconsistent documentation of complications and hearing results. Furthermore, language bias may have affected comprehensiveness, as only English‐language studies were included. These limitations collectively indicate a moderate to high overall risk of bias across the included studies, warranting cautious interpretation of the findings. Detailed results are shown in Table 3.

**TABLE 3:** Risk of bias assessment. [Color table can be viewed in the online issue, which is available at www.laryngoscope.com]

Study (author, year)	Selection (0–4)	Comparability (0–2)	Outcome (0–3)	Total score (0–9)
Rambousek (2012)	2	2	2	6
Thomeer (2011)	3	1	2	6
Dalchow (2007)	1	1	1	3
Magliulo (2007)	2	1	3	6
Fisch (2001)	3	1	3	7
Son (2024)	2	1	2	5
Alabdulqader (2021)	1	1	2	4
Gargula (2020)	1	1	0	2
Burggraaf (2018)	3	2	2	7
Ianella (2018)	2	1	3	6
Park (2014)	2	2	2	6
Magliulo (2013)	3	1	2	6
Schuknecht (1986)	3	0	1	4
Tange (1996)	3	0	1	4
Jahnke (2004)	3	0	3	6
Sarac (2006)	2	1	1	4
Kohan (2003)	1	1	3	5
Xu (2020)	1	1	1	3
Pitiot (2016)	1	2	2	5
Van Rompaey (2011)	3	2	1	6
Häusler (2007)	0	1	1	2
Kisilevsky (2009)	2	1	1	4
van der Rijt (2003)	2	0	2	4
De Leenheer (2003)	0	1	2	3
Ensink (1997)	1	1	2	4

*Note*: Red: high risk of bias; yellow: moderate risk of bias; green: low risk of bias.

## 4 Discussion

### 4.1 Terminological Considerations

In the current literature, the term “malleostapedotomy” is widely used to describe this surgical intervention. However, considering the procedural aspect of attaching the prosthesis to the malleus, we argue that the terminology should incorporate the suffix “‐malleopexy”, reflecting this distinctive feature. Alternative terms such as “stapedomalleopexy” or “vestibulomalleopexy” may more accurately convey the nature of the procedure. Nonetheless, given the extensive prevalence of “malleostapedotomy” in scientific literature, we have chosen to adopt this term in our article while acknowledging the potential advantages of a more precise nomenclature.

### 4.2 Surgical Indications

MS can be performed both in primary and revision surgeries. Almost every paper reported an “intraoperative findings” chart or section, describing more or less in detail the status of the ossicular chain, as checking the ossicular motility is a crucial phase of the stapedoplasty procedure. Surgical indications for MS are determined intraoperatively. Up to date, indications for performing primary MS include: stapes fixation associated with a missing, shortened or malformed long process of the incus [1, 3, 5, 8, 12, 16, 17, 19, 21]; stapes fixation associated with fixation [8, 15, 27, 28] or luxation [3, 10, 15, 27] of the incudostapedial joint; stapes fixation associated with a missing incus [17, 27]; stapes ankylosis associated with another congenital ossicular chain anomaly (Cremers class II) [10, 11]; stapes fixation associated with malleus fixation [1, 3, 5, 8, 11, 12, 15, 17, 18, 20, 27, 29]; stapes fixation associated with incus fixation [1, 3, 4, 5, 12, 16, 17, 28]; stapes fixation associated with incudo‐malleolar joint subluxation [5, 8, 18]; fixed footplate secondary to tympanosclerosis [8, 12]; accidental dislocation of the incus [1].

The same indications apply for MS in revision surgery. An MS revision procedure may be necessary due to persistent ABG, ABG degradation, or the onset of dizziness or vertigo. Intraoperative findings that could explain the onset of such symptoms include: displacement of the prosthesis, either in a single or both ends [3, 8, 9, 11, 16, 19, 20, 21]; malfunction of the prosthesis (too long, too short, twisted, fixed, loose) [3, 21]; granuloma/fibrous obstruction at the oval window [3, 21]; bony regrowth at the footplate [3]; failure of primary surgery due to an inappropriate check of the ossicular motility. For all these reasons, correct indications for MS require a thorough evaluation of the ossicular chain elements and their mobility. Some papers mentioned abnormalities of the tympanic membrane (perforations, atrophy, retractions, neomembrane) as a contraindication to MS [18].

### 4.3 Surgical Techniques

The MS procedure is conceptually similar to the classic stapedoplasty procedure, with the main difference being the placement of the prosthesis. MS can be performed under either local or general anesthesia.

Surgical techniques for MS have evolved over time. Fisch [3] introduced the term “Malleostapedotomy” when he made modifications to the technique previously proposed by Schuknecht, Bartley and Sheehy [1, 2].

The original technique involved lifting a tympanomeatal flap through a transcanal approach to expose the lateral face of the malleus. At this point, a wire was wrapped around the intermediate portion of the handle of the malleus, while the other end was placed into a footplate hole. Fisch proposed lifting a larger tympanomeatal flap to facilitate canaloplasty, allowing better exposure of the more cranial region of the malleus. Additionally, he performed a limited lifting of the flap, as the crimping of the prosthesis loop was meant to be as close as possible to the lateral malleal process. The aim of this change was to avoid excessive movement of the prosthesis inside the vestibule during maneuvers like Valsalva, sneezing, and rapid pressure changes (the excursion at the malleal umbo is wider than the excursion at the neck of the malleus). This explains why some authors suggest using a relatively long prosthesis inserted more deeply into the vestibule to reduce the risk of displacement at the medial end of the prosthesis [18]. Some authors also recommend interposing a small cartilage fragment between the loop and the tympanic membrane to prevent its exposure [7]. Adequate crimping to the malleus is essential to avoid resorption osteitis at the contact site [18]. The introduction of a dedicated prosthesis, with the possibility of bending its shaft, has made correct prosthesis placement easier.

In recent years, an endoscopic technique has also been described [6, 7]. This technique has proven to be an adequate alternative to the classic microscopic technique, providing better visualization of all structures, especially in patients with “difficult” anatomies, and a safer approach during stapedotomy. However, it is burdened by a quite difficult one‐hand maneuver, especially during prosthesis placement, and longer surgical times.

Only one study [27] focused on comparing the hearing outcomes between handle‐MS, the current technique, and neck‐MS. The latter technique seemed to have several advantages, such as a reduced risk of prosthesis displacement and extrusion, and the need to expose a smaller portion of the malleus during tympanomeatal flap elevation. Moreover, this technique showed similar hearing results compared to classic handle MS. Further investigations are needed to confirm whether the possible advantages suggested for surgical exposure and the reduced likelihood of prosthesis extrusion can be consistently demonstrated.

### 4.4 Types of Prostheses

Since the introduction of MS, significant progress has been made in prosthesis development. Early surgical experiences utilized wire‐fat prostheses or prefabricated wire‐loop prostheses [1]. The need for a specific type of prosthesis depends on several factors: a greater distance between the hook and the vestibule; the need for an adequate angle between the hook and the piston; good biocompatibility, as the hook is near the tympanic membrane.

While most studies included more than one specific piston prosthesis, some papers focused on the use of a particular type of prosthesis [4]. Crimping is one of the most challenging steps in the procedure; to simplify it, some authors have described the use of self‐crimping prostheses [4, 6, 14]. The locking mechanism of the first auto‐crimping prostheses was mechanical [4], whereas other prostheses utilized material features (such as elastic memory) or heat‐activated mechanisms to facilitate attachment to the malleus [5, 29]. Most of the used prostheses had the shape of a piston linked to a hook, with an adjustable angle between its segments; some other prostheses presented an articulation between the rod and the hook. The piston should always be positioned perpendicularly to the footplate. The shape of the prosthesis influences the surgical technique. The first type of prosthesis requires positioning the shaft into the footplate hole first, followed by malleopexy. Articulated prostheses allow this sequence to be inverted, reducing surgical times and avoiding the need for piston length measurements [4]. Nowadays, the most commonly used prostheses are made of metals (platinum, titanium, steel, Nitinol) alone or in combination with other materials (usually fluoroplastic, as Teflon). This latter allows the prosthesis to be trimmed to the desired length.

### 4.5 Hearing Results and Complications

Almost every paper reported audiological results, although there is significant heterogeneity in how these results were presented. Some studies reported the preoperative and postoperative hearing thresholds in aggregate, while others lacked this kind of information. Almost all studies present the results as the ABG closure, meaning the difference between the preoperative and postoperative ABG. Some studies considered an ABG closure within 10 dB as successful [7].

We reported an excellent result in ABG closure in 40.4% of cases and a good result in 82.4% of cases. We employed weighted percentages, based on the number of patients per study, to mitigate potential reporting bias. However, these findings should be interpreted with caution, given the heterogeneity in audiometric reporting methods, follow‐up durations, and the overall risk of bias among the included studies.

Some considerations are needed. Firstly, there was no homogeneity in the number of frequencies evaluated. Some studies considered only the 0.5, 1 and 2 kHz frequencies, while some others included additional frequencies such as 3 and 4 kHz. Some studies presented audiological results according to the guidelines recommended by the American Academy of Otolaryngology Committee on Hearing and Equilibrium, which advises recording pure‐tone averages for air conduction and bone conduction using frequencies of 0.5, 1, 2 and 3 kHz, then calculating the difference between the average preoperative and postoperative ABGs [30].

Secondly, there was no agreement on the timing of follow‐up. Some studies evaluated the postoperative thresholds at 6 weeks [9], 3 months [11], 6 months [6], and others at 12 months [27, 28]; some other studies did not specify the timing of the follow‐up. It is important to note that the ABG closure alone may lead to an underestimation of sensorineural hearing loss. For this reason, some studies separately reported the cases with worsened bone conduction thresholds, even if there was an ABG closure.

Several studies have attempted to compare outcomes between MS and IS, but the evidence remains inconclusive. Son et al. reported comparable audiological results between endoscopic MS and endoscopic IS [27]. Fisch observed a significant advantage of MS in achieving ABG closure within 10 dB, whereas outcomes were similar between the two techniques when considering closure within 20 dB [3]. Kisilevsky found that IS was associated with significantly better ABG closure than MS, although this result was limited to patients with congenital hearing loss [23]. Thomeer compared IS and MS in terms of audiological outcomes and found no statistically significant differences between the two groups [10]. Similarly, Kohan stated that “the results are comparable with standard incus to oval window reconstructive techniques,” although the study involved a small sample and no formal statistical analysis was presented [19].

In addition to functional outcomes, Fisch also provided comparative data on postoperative complications. In that series, the rate of sensorineural hearing loss was approximately 1.8% after MS and 6.7% after IS, with no cases of dead ear reported in either group [3].

Overall, the available studies are few in number, heterogeneous in design, and often limited by small sample sizes, diverse surgical techniques, and variable follow‐up durations. Consequently, while preliminary evidence suggests that both MS and IS can achieve satisfactory functional outcomes, with a possibly lower rate of sensorineural complications after MS, no definitive conclusions can be drawn regarding the superiority of one technique over the other.

A recent meta‐analysis examined the audiological outcomes of endoscopic and microscopic stapes surgery in patients with stapes fixation. The data indicate that 73.3% of patients achieved an excellent result (ABG closure < 10 dB), while 93.7% attained a good result (ABG closure < 20 dB) [31]. Another meta‐analysis focused solely on revision cases of stapes surgery, reporting an ABG closure of less than 10 dB in 57.2% of cases, and less than 20 dB in 79% of cases [32].

Almost every study included a “complications” section. Most complications are the same as those for IS, except for prosthesis extrusion, which may indicate the need for reintervention. Other complications include vertigo and dizziness, chorda tympani injuries/disgeusia, sensorineural hearing loss, and dead ear. Some studies report a decrease in bone conduction over specific frequencies, specifying the absence of significant sensorineural hearing loss. However, the cut off between these conditions was determined arbitrarily.

### 4.6 Other Viable Procedures

In cases of stapes revision surgery and erosion of the long process of the incus, some authors have suggested using bone cement. This synthetic material can be used both to fix the prosthesis on the long process remnant and to lengthen the long apophysis of the incus. Pitiot and others have recommended using hydroxyapatite bone cement to fix the prosthesis to the incus as a first‐line strategy, while advising against using this material to lengthen the long process of the incus [21]. TORP could also be useful; however, it showed worse audiological results and should be considered a last‐resort strategy when bone cement and MS are not feasible. It is important to note that the introduction of bone cement for ossicular reconstruction is a relatively recent innovation, and information about the long‐term stability of the reconstruction is not yet available. Hearing aids could be another feasible alternative.

## 5 Conclusion

Based on literature data MS is a viable option in cases of primary and revision surgery in patients with stapes fixation. The surgical technique has evolved over time, thanks to the introduction of newer prostheses and, more recently, endoscopy. Results are slightly worse than the traditional technique of incus‐anchoring stapedoplasty; nevertheless, it represents a safe and valid procedure when this latter technique is not viable.

## Conflicts of Interest

The authors declare no conflicts of interest.

## Supporting information


**File S1:** Modified Newcastle–Ottawa Scale (NOS) used for risk of bias assessment.

## Data Availability

The data used in this study were obtained from previous studies that are easily accessible online; all such studies are cited in the References section.
