# Supplementary material for: Malleostapedotomy in Patients With Stapes Fixation: A Systematic Review
Source: Laryngoscope. 2025 Nov 13;136(4):1601–13. doi: 10.1002/lary.70232 (PMC12993101; doi:10.1002/lary.70232)
Supplement: Supplementary file 1 — File S1: Modified Newcastle–Ottawa Scale (NOS) used for risk of bias assessment. [file LARY-136-1601-s001.docx]

# Risk of Bias Assessment – Modified Newcastle–Ottawa Scale (NOS)

## 1. Selection (0–4 points)

Evaluates how patients were selected and how representative they are.

• Representativeness of the cohort
 - 1 = consecutive or representative of the target population
 - 0 = unclear or restricted selection

• Clearly defined inclusion criteria
 - 1 = yes, specified (e.g., diagnosis, surgical indications)
 - 0 = no, absent or vague

• Standardized diagnostic methodology
 - 1 = yes (use of audiometric criteria, imaging, clearly defined protocols)
 - 0 = no

• Adequate sample size (≥20 patients)
 - 1 = yes
 - 0 = no

## 2. Comparability (0–2 points)

Evaluates the control of potential confounding factors.

• Control of relevant clinical variables (age, prosthesis type, surgical technique, surgeon’s experience)
 - 1 = yes, reported and considered
 - 0 = no

• Use of comparable groups or clearly defined subgroups (e.g., comparison between techniques, clear stratification)
 - 1 = yes
 - 0 = no

## 3. Outcome/Exposure (0–3 points)

Evaluates the quality of outcome measurement.

• Clearly defined outcome assessment (e.g., standardized audiometry, clear definition of ABG closure and tested frequencies)
 - 1 = yes
 - 0 = no

• Adequate and reported follow-up (e.g., ≥6 months or clearly specified)
 - 1 = yes
 - 0 = no

• Completeness of data and handling of losses to follow-up
 - 1 = yes, described and addressed
 - 0 = no

## Total Score and Risk of Bias Classification

• 0–3 points = High risk of bias
• 4–6 points = Moderate risk of bias
• 7–9 points = Low risk of bias
